# From policy to action: how to operationalize the treatment for all agenda

**DOI:** 10.7448/IAS.19.1.21185

**Published:** 2016-12-16

**Authors:** Francesca Celletti, Jennifer Cohn, Catherine Connor, Stephen Lee, Anja Giphart, Julio Montaner

**Affiliations:** 1 Elizabeth Glaser Pediatric AIDS Foundation, Washington, DC, USA; 2 Division of Infectious Diseases, University of Pennsylvania School of Medicine, Philadelphia, PA, USA; 3 Faculty of Medicine, University of British Columbia, Vancouver, BC, Canada

## Introduction

The 2015 World Health Organization (WHO) guidelines recommend antiretroviral treatment (ART) for all people living with HIV, regardless of CD4 count. Treatment for all (TfA) represents a significant step towards meeting the ambitious United Nations’ 90–90–90 target by 2020 and ending AIDS by 2030 [1]. Achieving these goals will require focused, multilateral efforts. Globally, only 54% of people living with HIV are now aware of their HIV status and only 41% of adults and 32% of children diagnosed with HIV are on ART [2]. It is, therefore, clear that we are far from realizing the full benefits of ART, and substantial efforts will be needed to meet the 90–90–90 target.

To move from policy to action and achieve TfA, we urgently need to renew our operational agenda. We must rapidly expand access to and uptake of comprehensive HIV services while introducing efficiencies and new models of care that will enable overburdened health systems to sustain current patients while accelerating intake of new patients. This strategy must be based on realities on the ground and be designed to 1) make care simpler and more accessible, 2) create rational models of care that are responsive to patients’ needs and 3) identify and implement transformative tools to reach TfA.

This article proposes the development and adoption of a differentiated care model as a practical approach to reach TfA and accelerate progress towards the 90–90–90 target.

### Current knowledge

#### HIV testing

Efficient approaches to HIV testing, tailored to client values and preferences, have increased the detection of HIV infection. For example, several large community-based HIV testing studies have shown acceptance rates of 80% or greater, linkage to care of 80% and eventual treatment initiation of 73% [3]. The Accept project, a cluster-randomized trial, demonstrated that widespread community mobilization and provision of mobile testing services were associated with a 14% fall in HIV incidence [4]. Two large multi-disease prevention campaigns, in Kenya and Uganda, showed high uptake of HIV testing [5]. In a Malawian study, 75% of people took an annual self-test, and more than half of those diagnosed were linked to HIV care [6].

Addressing the testing needs of vulnerable and priority populations may also result in increased testing yield and linkage to care. Men – who access the health system less frequently than women – are tested in higher numbers when HIV testing is offered at work sites as compared to HIV testing clinics (e.g. 51.1% vs. 19.2%) [7, 8]. And, although the evidence is limited, provider-initiated testing and counselling of children in key health service entry points have shown an average yield of 16.6%, with paediatric inpatient units showing 22.5% [9].

#### HIV treatment and retention

A number of evidence-based interventions have demonstrated success in simplifying ART initiation and treatment. Nurse-initiated and managed ART has been shown to be as or more effective at reducing mortality and achieving undetectable viral load when compared with standard physician-initiated ART care [10, 11]. The 2015 WHO guidelines recommend spacing visits once every three to six months and dropping CD4 testing for patients who are stable and virologically suppressed on ART [12–14]. Members of community-based adherence clubs and community ART groups, and recipients of community ART distribution, achieve equal or higher levels of retention in care and viral load suppression as compared to facility-based patients [15–18]. Routine viral load monitoring can be used in a cost-effective way to identify stable, virologically suppressed patients for whom care can be streamlined, including through community-managed care [19]. Paediatric patients also do well in decentralized care models, with one cohort study of community-based versus facility-based care showing no significant difference in survival rates and improved retention for community-based versus facility-based care (94.8% vs. 84.7%) [20]. These interventions also lower costs to the healthcare system [21] and will simplify management, help normalize the lives of patients on ART and make use of available resources more efficiently.

As a significant loss to follow-up (32–54%) occurs in the period before ART initiation [22, 23], reducing pre-ART time has helped increase retention. New data show that same-day initiation results in higher retention [24, 25]. Other simple interventions improve retention after ART initiation. Home-based visits by community health workers soon after ART initiation can help decrease early loss to follow-up after ART initiation [26]. Reminders sent via short message service (SMS) to cell phones result in significantly higher adherence for patients on ART [27, 28].

#### Treatment for all

The TfA approach is not new. Since 2003, the Government of British Columbia has progressively expanded access to ART. In 2009, the province formalized a universal, fully funded TfA policy, known as Seek and Treat for Optimal Prevention of HIV/AIDS. This policy has been associated with marked and steady decreases in AIDS-related incidence, morbidity and mortality [29]. Similarly, the University of California, San Francisco/San Francisco General Hospital RAPID programme offers immediate access to ART on the same day as HIV diagnosis. Under this initiative, ART uptake has quadrupled and viral load suppression has doubled [30]. Encouragingly, comparable trends are emerging in limited resource settings. A study in KwaZulu-Natal, South Africa, has reported that an increase in ART coverage of 1% was associated with a 1% decrease in new HIV infections [31]. In programmes like these, even when initiating asymptomatic patients at higher CD4 counts, adherence levels remain high [32].

Another promising TfA strategy is Option B+, universal offer of lifelong ART to all HIV-positive pregnant and lactating women. The Elizabeth Glaser Pediatric AIDS Foundation has supported the rollout of Option B+ programmes in 12 countries since 2011 and shown that initiating ART regardless of CD4 count is feasible and effective in resources-constrained settings. It has also demonstrated the need to change the current system to enable earlier identification of patients and retention of large numbers of healthy patients in quality care. In fact, many women still presented late, with HIV Stage III or IV disease. Further, only 74% of women on ART (excluding transfer outs) were retained in care at 12 months in Malawi [33].

### Differentiated care packages to reach Treatment for All

TfA will require a comprehensive package of differentiated testing, care and treatment fit to different contexts and needs of different patient populations, including children and adolescents. Therefore, we must support decentralization and simplification at the individual and programmatic level for stable, suppressed individuals and for well-functioning programmes. We must also employ patient- and programme-level data to identify and act upon unstable, poorly functioning and immature programmes.

**Figure 1:**
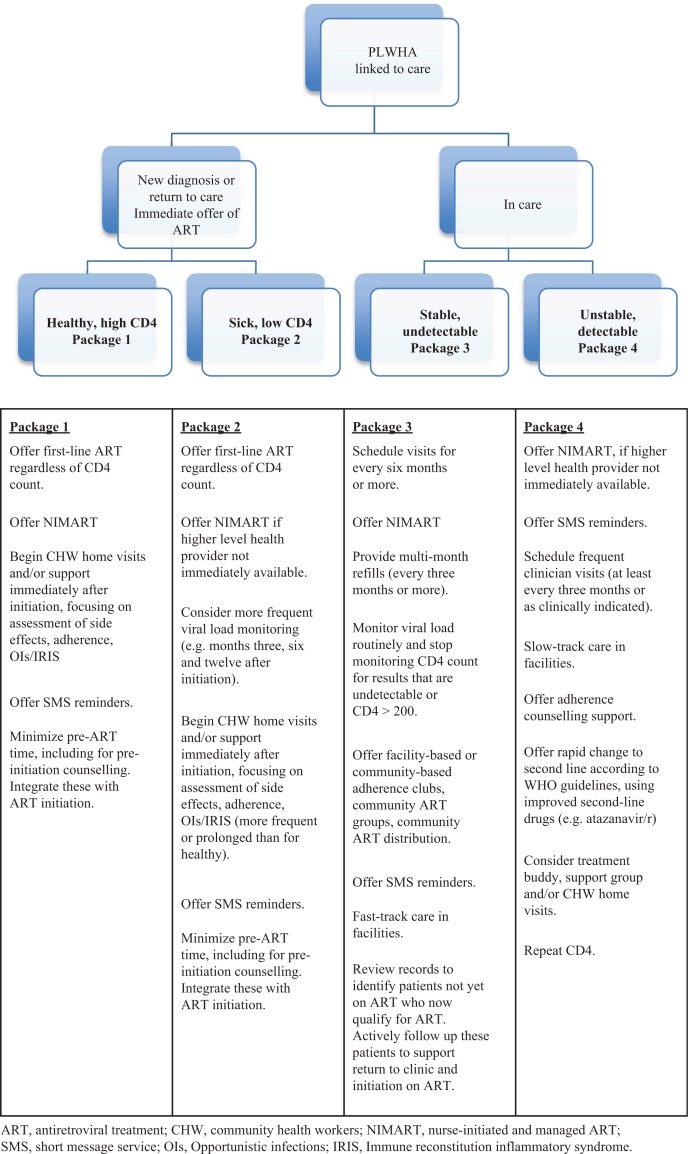
Proposed standard package of interventions for differentiated care.

A differentiated package of care should include proven interventions, tools and models to generate the greatest efficiencies and ensure maximum returns from limited healthcare resources. It will also be critical that these interventions are not only acceptable to but also desired by patients and communities. The proposed package (Figure 1) highlights breakpoints that may be used to support differentiated care. The interventions described may be considered a basic package; new tools and other innovative models of care should be considered, piloted and – if effective – scaled up. These might include detecting acute HIV infection using point-of-care viral load monitoring; collecting viral load samples at very decentralized facilities via dried blood spots, with results returned by SMS; or adopting optimized treatment, including long-acting injectable ART for key populations.

Use of standard packages to optimize differentiated care must be field-tested for feasibility and impact on the achievement of TfA. Successful implementation of these packages will also be dependent on 1) the placement of policies that enable task shifting; 2) the development of metrics that reflect progress towards the 90–90–90 and assess the impact of the differentiated package of care; 3) the existence of sufficient programmatic infrastructure, including procurement and supply chains; and 4) support of sentinel sites to monitor transmitted and acquired drug resistance and to inform future first-line ART [34] – especially in light of growing non-nucleoside reverse-transcriptase inhibitors [35] resistance and higher-than-expected tenofovir disoproxil fumarate [36] resistance.

As the TfA approach progresses globally, HIV epidemiology will evolve, resulting in larger cohorts of stable, virologically suppressed patients and a decrease in HIV incidence. A more simplified approach for the management of these stable cohorts will be possible, and a different strategy and treatment approach may be required. Both the health system and the patients in these stable cohorts will benefit from a pathway that includes fewer clinic visits, streamlined and community-based drug distribution and reduced laboratory monitoring requirements. Over time, the authors feel that normalizing TfA, identifying and treating patients earlier in the disease to keep them healthy and simplifying care by allowing community-based care will help reduce the stigma associated with HIV. Although TfA will generate long-term benefits and reduce costs overall, the strategy will initially require increased political and financial capital [37].

## Conclusions

The WHO's 2015 guidelines and the differentiated package of care are critical building blocks for designing and implementing a TfA strategy, but more must be done. Countries and operational partners should rapidly implement TfA while designing and documenting operational research to identify best practices, improve case finding and reach treatment success while simplifying, decentralizing and streamlining care to improve the rational use of resources. Communities, including populations living with HIV, service providers and national and global policymakers, will need to

- Accept that excellent care can be algorithmic and performed by healthcare workers with limited training
- Allow stable, undetectable patients in treatment to manage their own care by accessing community-based care and reducing clinic visits
- Assess and pilot innovative tools and models of care
- Exchange CD4 monitoring for viral load monitoring
- Normalize and integrate ART through service provider education on the benefits of TfA and multi-disease programming that includes ART initiation and support alongside other primary health interventions such as hypertension screening or blood glucose testing
- Support operational research on various packages of differentiated care to better define the impact, feasibility and cost-effectiveness of various models and expand the evidence supporting this promising model of care
- Provide appropriate investment in such models of care to reach TfA

To virtually end AIDS by 2030, a collective investment has to take place now, using our most effective tools, policies, strategies and resources to operationalize TfA.

## References

[1] UNAIDS 90-90-90: an ambitious treatment target to help end the AIDS epidemic.

[2] UNAIDS AIDS by the numbers 2015.

[3] Suthar AB, Ford N, Bachanas PJ, Wong VJ, Rajan JS, Saltzman AK (2013). Towards universal voluntary HIV testing and counselling: a systematic review and meta-analysis of community-based approaches. PLoS Med.

[4] Coates TJ, Kulich M, Celentano DD, Zelaya CE, Chariyalertsak S, Chingono A (2014). Effect of community-based voluntary counselling and testing on HIV incidence and social and behavioural outcomes (NIMH Project Accept; HPTN 043): a cluster-randomised trial. Lancet Glob Health.

[5] Chamie G, Kwarisiima D, Clark TD, Kabami J, Jain V, Geng E (2014). Uptake of community-based HIV testing during a multi-disease health campaign in rural Uganda. PLoS One.

[6] Choko AT, MacPherson P, Webb EL, Willey BA, Feasy H, Sambakunsi R (2015). Uptake, accuracy, safety, and linkage into care over two years of promoting annual self-testing for HIV in Blantyre, Malawi: a community-based prospective study. PLoS Med.

[7] Corbett EL, Dauya E, Matambo R, Cheung YB, Makamure B, Bassett MT (2006). Uptake of workplace HIV counselling and testing: a cluster-randomised trial in Zimbabwe. PLoS Med.

[8] Collier AC, Van der Borght SF, Rinke de Wit T, Richards SC, Feeley FG (2007). A successful workplace program for voluntary counseling and testing and treatment of HIV/AIDS at Heineken, Rwanda. Int J Occup Environ Health.

[9] Cohn J, Whitehouse K, Tuttle J, Lueck K, Tran T (2016). Pediatric HIV testing beyond the PMTCT context: a systematic review and meta-analysis. Lancet HIV.

[10] Brennan AT, Long L, Maskew M, Sanne I, Jaffray I, MacPhail P (2011). Outcomes of stable HIV-positive patients down-referred from a doctor-managed antiretroviral therapy clinic to a nurse-managed primary health clinic for monitoring and treatment. AIDS.

[11] Shumbusho F, van Griensven J, Lowrance D, Turate I, Weaver MA, Price J (2009). Task shifting for scale-up of HIV care: evaluation of nurse-centered antiretroviral treatment at rural health centers in Rwanda. PLoS Med.

[12] WHO Consolidated guidelines on the use of antiretroviral drugs for treating and preventing HIV infection 2016: Recommendations for a public health approach.

[13] Bemelmans M, Baert S, Goemaere E, Wilkinson L, Vandendyck M, van Cutsem G (2014). Community-supported models of care for people on HIV treatment in sub-Saharan Africa. Trop Med Int Health.

[14] Ford N, Stinson K, Gale H, Mills EJ, Stevens W, Pérez González M (2015). CD4 changes among virologically suppressed patients on antiretroviral therapy: a systematic review and meta-analysis. J Int AIDS Soc.

[15] Bemelmans M, van den Akker T, Ford N, Philips M, Zachariah R, Harries A (2010). Providing universal access to antiretroviral therapy in Thyolo, Malawi through task shifting and decentralization of HIV/AIDS care. Trop Med Int Health.

[16] Decroo T, Telfer B, Biot M, Maïkéré J, Dezembro S, Cumba LI (2011). Distribution of antiretroviral treatment through self-forming groups of patients in Tete Province, Mozambique. J Acquir Immune Defic Syndr.

[17] Zachariah R, Teck R, Buhendwa L, Fitzerland M, Labana S, Chinji C (2007). Community support is associated with better antiretroviral treatment outcomes in a resource-limited rural district in Malawi. Trans R Soc Trop Med Hyg.

[18] Selke HM, Kimaiyo S, Sidle JE, Vedanthan R, Tierney WM, Shen C (2010). Task shifting of antiretroviral delivery from health care workers to persons living with HIV/AIDS: clinical outcomes of a community-based program in Kenya. J Acquir Immune Defic Syndr.

[19] Phillips A, Shroufi A, Vojnov L, Cohn J, Roberts T, Ellman T (2015). Sustainable HIV treatment in Africa through viral load-informed differentiated care. Nature.

[20] Massavon W, Barlow-Mosha L, Mugenyi L, McFarland W, Gray G, Lundin R (2014). Factors determining survival and retention among HIV-infected children and adolescents in a community home-based care and a facility-based family-centred approach in Kampala, Uganda: a cohort study. ISRN AIDS.

[21] Marseille E, Kahn JG, Pitter C, Bunnell R, Epalatai W, Jawe E (2009). The cost effectiveness of home-based provision of antiretroviral therapy in rural Uganda. Appl Health Econ Health Policy.

[22] Mugglin C, Estill J, Wandeler G, Bender N, Egger M, Gsponer T (2012). Loss to programme between HIV diagnosis and initiation of antiretroviral therapy in sub-Saharan Africa: systematic review and meta-analysis. Trop Med Int Health.

[23] Larson B, Brennan A, McNamara L, Long L, Rosen S, Sanne I (2010). Lost opportunities to complete CD4+ lymphocyte testing among patients who tested positive for HIV in South Africa. Bull World Health Organ.

[24] Thumath M, Moore D, Hull M, Brownrigg B, Sandstra I, Ogilvie G Implementation of a rapid referral pathway to HIV treatment for gay men and MSM diagnosed with acute HIV-infection in sexual health clinics in British Columbia.

[25] Rosen S, Maskew M, Fox M, Nyoni C, Mongwenyaya C, Malete G (2016). Initiating antiretroviral therapy for HIV at a patient's first clinic visit: the rapIT randomized controlled trial. PLoS Med.

[26] Bärnighausen T, Chaiyachati K, Chimbindi N, Peoples A, Haberer J, Newell ML (2011). Interventions to increase antiretroviral adherence in sub-Saharan Africa: a systematic review of evaluation studies. Lancet Infec Dis.

[27] Lester RT, Ritvo P, Mills EJ, Kariri A, Karanja S, Chung MH (2010). Effects of a mobile phone short message service on antiretroviral treatment adherence in Kenya (WelTel Kenya1): a randomised trial. Lancet.

[28] Pop-Eleches C, Thirumurthy H, Habyarimana JP, Zivin JG, Goldstein MP, de Walque D (2011). Mobile phone technologies improve adherence to antiretroviral treatment in a resource-limited setting: a randomized controlled trial of text message reminders. AIDS.

[29] Clarke C, Cheng T, Barrios R, Reims KG, Steinbock CM, Thumath M (2015). Implementation of HIV treatment as prevention^®^strategy in 17 Canadian sites: immediate and sustained outcomes from a 35-month quality improvement collaborative. BMJ Qual Saf.

[30] Pilcher C, Hatano H, Dasgupta A, Jones D, Torres S, Calderon F Providing same day, observed ART to newly diagnosed HIV+ outpatients is associated with improved virologic suppression.

[31] Tanser F, Bärnighausen T, Grapsa E, Zaidi J, Newell ML (2013). High coverage of ART associated with decline in risk of HIV acquisition in rural KwaZulu-Natal, South Africa. Science.

[32] Jan V, Byonanebye DM, Amanyire G, Kwarisiima D, Black D, Kabami J (2014). Successful antiretroviral therapy delivery and retention in care among asymptomatic individuals with high CD4+ T-cell counts above 350 cells/µl in rural Uganda. AIDS.

[33] UNAIDS Malawi AIDS response progress report 2015.

[34] The TenoRes Study Group (2016). Global epidemiology of drug resistance after failure of WHO recommended first-line regimens for adult HIV-1 infection: a multicentre retrospective cohort study. Lancet Infect Dis.

[35] Baxter JD, Dunn D, White E, Sharma S, Gerretti AM, Kozal MJ (2015). Global HIV-1 transmitted drug resistance in the INSIGHT Strategic Timing of AntiRetroviral Treatment (START) Trial. HIV Medicine.

[36] Manasa J, Danaviah S, Lessells R, Elshareef M, Tanser F, Wilkinson E (2016). Increasing HIV-1 drug resistance between 2010 and 2012 in adults participating in population-based HIV surveillance in rural KwaZulu-Natal, South Africa. AIDS Res Hum Retroviruses.

[37] UNAIDS A new investment framework for the global AIDS response.

